# Assessment and clinical management of bone disease in adults with eating disorders: a review

**DOI:** 10.1186/s40337-017-0172-0

**Published:** 2017-12-04

**Authors:** Anne Drabkin, Micol S. Rothman, Elizabeth Wassenaar, Margherita Mascolo, Philip S. Mehler

**Affiliations:** 1 Denver Health and Hospital Authority, 660 Bannock MC 4000, Denver, CO 80204 USA; 2 University of Colorado Hospital, Anschutz Medical Campus, 13001 E 17th Pl, Aurora, CO 80045 USA; 3 Eating Recovery Center, 7351 E. Lowry Blvd. Suite 200, Denver, CO 80230 USA

**Keywords:** Bone mineral density, Osteopenia, Osteoporosis, Anorexia nervosa, Malnutrition, Premenopausal

## Abstract

**Aim:**

To review current medical literature regarding the causes and clinical management options for low bone mineral density (BMD) in adult patients with eating disorders.

**Background:**

Low bone mineral density is a common complication of eating disorders with potentially lifelong debilitating consequences. Definitive, rigorous guidelines for screening, prevention and management are lacking. This article intends to provide a review of the literature to date and current options for prevention and treatment.

**Methods:**

Current, peer-reviewed literature was reviewed, interpreted and summarized.

**Conclusion:**

Any patient with lower than average BMD should weight restore and in premenopausal females, spontaneous menses should resume. Adequate vitamin D and calcium supplementation is important. Weight-bearing exercise should be avoided unless cautiously monitored by a treatment team in the setting of weight restoration. If a patient has a Z-score less than expected for age with a high fracture risk or likelihood of ongoing BMD loss, physiologic transdermal estrogen plus oral progesterone, bisphosphonates (alendronate or risedronate) or teriparatide could be considered. Other agents, such as denosumab and testosterone in men, have not been tested in eating-disordered populations and should only be trialed on an empiric basis if there is a high clinical concern for fractures or worsening bone mineral density. A rigorous peer-based approach to establish guidelines for evaluation and management of low bone mineral density is needed in this neglected subspecialty of eating disorders.

## Plain English summary

Young adults with eating disorders can develop osteoporosis, or fragile bones, which can cause lifelong debilitating consequences. Despite its high prevalence, general guidelines for diagnosis and treatment are lacking and further collaboration is needed. Some current osteoporosis medications may have severe side effects or cause birth defects in pregnant women and thus require special scrutiny. Currently, weight restoration, resumption of a regular menstrual period in women and ensuring adequate vitamin D and calcium levels are the mainstays of therapy. This review summarizes the current literature, outlines best practice recommendations and suggests areas for improvement in the field to better help these patients in the future.

## Background

Eating disorders are becoming more common in the United States and currently affect approximately 20 million women and 10 million men [1]. They are defined by the Diagnostic and Statistical Manual of Mental Disorders, Fifth Edition (DSM–V) as “a persistent disturbance of eating or eating-related behavior that results in altered consumption or absorption of food and that significantly impairs physical health or psychosocial functioning” [2]. Eating disorders represent a unique intersection of brain-based disorders that are associated with catastrophic physical consequences. The lifetime prevalence of anorexia nervosa (AN) is estimated to be 0.9%, for bulimia nervosa 1.5%, and for binge eating disorder 3.5% among women; with a prevalence of 0.3%, 0.5%, and 2.0% respectively among men [3–5]. AN is associated with an extraordinarily elevated premature mortality rate estimated between 4.1–5.86 per 1000 person years [6, 7], most commonly due to suicide or sudden cardiovascular complications [6, 8].

Many consequences of AN can be reversed with weight restoration and resumption of normal eating behaviors. However, other complications, such as low bone mineral density (BMD), can persist for decades after disease resolution [9, 10] and cause lifelong debilitation. Deterioration of bone health can be seen with AN-induced malnutrition, affecting over 90% of malnourished inpatients [11]. It is an insidious consequence of AN given its lack of clinical symptoms, but is associated with prolonged increased fracture risk [9, 10, 12–14]. Fragility fractures in the malnourished patient with AN can prove detrimental to young individuals and can lead to permanent disability. Prompt evaluation and management of low BMD is crucial in preventing fractures in this susceptible population.

Despite the prevalence of low BMD in eating disorders, clear definitions and treatment guidelines are lacking. This is primarily due to an unclear approach to diagnosis and management of low BMD in young patients in general, but also due to limited data in the eating disorder population. A rigorous peer-based approach to establishing guidelines is further needed in this neglected area of eating disorders.

## Methodology

Studies published in the English language between 1996 and 2016 were searched in PubMed. We used keywords for the search including “anorexia nervosa” and “bone density” and then manually selected relevant papers based on the number of included subjects, journal impact factor and participant age range. Treatment-specific articles for this patient population were searched using additional keywords such as “premenopausal” and “treatment” and randomized controlled trials were favored for review. Several articles were referenced prior to 1996 that provided essential baseline data for this population that were not found in the abovementioned search criteria.

### Definition

The World Health Organization (WHO) and International Society of Clinical Densitometry (ISCD) clearly define “osteopenia” and “osteoporosis” in postmenopausal women and men over the age of 50 [15, 16]. However, given the general lack of longitudinal data, these definitions, treatment guidelines or use of the Fracture Risk Assessment Tool (FRAX) cannot be applied to the typically younger patients with AN [17]. Bone mineral density scores, based on dual-energy X-ray absorptiometry (DXA) scans, are classified as osteoporosis if the T-score is below or equal to 2.5 standard deviations below the norm (T-score ≤ −2.5). Osteopenia is defined as a T-score between 1.0 and 2.5 standard deviations below the norm (T-score − 1.0 to −2.5). In the premenopausal population and in men under age 50, Z-scores are preferred. T-scores compare a patient’s BMD to that of a healthy young adult at peak bone mass, whereas Z-scores use age and sex-matched comparisons [15, 18]. Z-scores less than 2 standard deviations below the norm (≤ −2.0) are defined as “below the expected range for age” and Z-scores greater than −2.0 meet the “expected range for age” [15, 16]. Patients with Z-scores less than −2.0, with a fracture risk or a continued cause of bone loss may be classified as having osteoporosis.

In contrast, the International Osteoporosis Foundation (IOF), recommends the use of T-scores in patients aged 20 to 50 years old and classifies osteoporosis as a T-score less than −2.5 [18]. At the current time, clinicians must ultimately use the nomenclature with which they are most familiar and make consistent therapeutic decisions based on their best clinical judgment and available data.

### Prevalence

Reduced BMD is frequently seen in patients with eating disorders [9, 11, 15, 19–22]. The data consistently report evidence of low BMD in patients with AN, which includes anorexia nervosa restricting subtype (AN-R) and anorexia nervosa binge/purge subtype (AN-BP), but the data are varied in regards to its prevalence in bulimia nervosa (BN) and avoidant restrictive food intake disorder (ARFID) previously known as eating disorder not otherwise specified (ED-NOS per DSM-IV) [11, 15, 19, 23]. In one study, greater than 90% of patients with AN had a T-score less than −1.0 and 38% had a T-score less than −2.5 [11]. There are two recent meta-analyses which evaluated the association between eating disorder subtype and bone density [19, 21]. In the meta-analysis by Robinson et al., 27 studies were reviewed that reported significantly lower BMD of the spine in AN and BN patients compared to healthy controls. The meta-analysis by Solmi et al. reviewed 57 studies to find that a significantly lower BMD existed between AN and healthy controls but not between BN and controls. Fracture risk in patients with ARFID shows mixed results in the literature [10, 21]. In a study reviewing all subtypes of severely malnourished eating disorder patients requiring inpatient hospitalizations (average body mass index 13.0), 83% of all patients had low BMD. However, this study did not differentiate the prevalence of bone disease by eating disorder subtype [24].

### Pathophysiology, bone abnormalities and fracture risk in anorexia nervosa

The etiology of decreased bone strength in AN is multifactorial and still under evaluation. Some patients with AN have well-established risk factors for low BMD, such as cigarette smoking or alcohol consumption [15]. Other comorbidities including gastrointestinal malabsorption, hyperthyroidism, renal or liver disease can be seen in patients with AN, which places them at an increased risk for low bone density and may warrant a referral to an appropriate specialist [25]. Additionally, medications such as corticosteroids, some diuretics, proton pump inhibitors (PPIs), selective serotonin reuptake inhibitors (SSRIs) or antiepileptic drugs (AEDs) such as valproic acid and carbamazepine [26] may also contribute to low BMD. However, as many patients with AN have a clinical benefit from the abovementioned medications, the clinician must use caution and weigh the risks and benefits of discontinuing or altering doses.

Many pathophysiologic changes leading to low BMD in patients with AN are closely linked to decreased body mass index (BMI) and malnutrition [9–11, 15, 19–22]. Broadly speaking, a decrease in BMI alters body composition and leads to multiple physiologic and adaptive hormonal changes. Downregulation in the hypothalamic-pituitary-gonadal axis leads to decreased estrogen and testosterone levels [27–30]. Women and adolescent girls with AN have decreased levels of estrogen compared to healthy controls, and a study in adolescent boys demonstrated decreased serum testosterone [29–31]. This can cause clinical symptoms such as oligo-amenorrhea in females and decreased libido in males. It should be noted that not all women with AN have ovulatory dysfunction or experience oligo-amenorrhea and women can ovulate without menstruation. Amenorrhea is, therefore, no longer required for the diagnosis of AN per the new DSM-V criteria [2]. Estrogen and testosterone affect bone turnover either directly or indirectly via inflammatory cytokines and stimulation of osteoclast formation, function and survival via the receptor activator of nuclear factor kappa-B ligand pathway (RANKL).

Hypercortisolism is also seen in patients with AN, likely due to many physiologic changes including an increased basal cortisol secretion, increased steroid pulse frequency and reduced renal cortisol clearance [27, 32]. Similar to patients taking exogenous corticosteroids, hypercortisolism can lead to lower lean mass of the extremities and low BMD via decreased bone formation and increased bone resorption [33, 34].

Other hormonal shifts affected by malnutrition include a decrease in insulin-like growth factor-1 (IGF-1), leptin, insulin and oxytocin along with growth hormone (GH) resistance [35–38]. An increase in peptide YY and adiponectin, plus ghrelin resistance, is also noted and their exact relationships to bone disease are currently being studied [37, 39].

Changes in body composition in AN include a decrease in lean mass and brown adipose tissue (BAT) and an increase in bone marrow adipose tissue [40, 41]. In a small study in 2012, the presence of BAT was associated with higher BMD and only 20% of patients with AN had BAT compared to 80% of healthy comparisons [41]. Muscular pull on attached bone has an anabolic effect on bone growth and a decrease in lean mass is known to cause low BMD in AN [31, 42]. Similar to the long-term effects of malnutrition on bone, AN may also cause longstanding muscular impairment [43].

Bone strength is most frequently reflected by bone mineral density measurements but is also influenced by other factors such as bone geometry, microarchitecture, bone turnover and degree of mineralization. A DXA scan is the most common and accepted method of determining bone density given its ease of use and relatively low cost. Low bone mineral density is a common consequence of AN and has been seen repeatedly in the literature [13, 30, 44]. In a study of 75 women with active AN, an annual BMD decline of 2.6% at the spine and 2.4% at the hip was observed [45].

Bone geometry refers to the size and shape of a bone and can be analyzed with hip structural analysis on DXA. Several studies have noted impaired bone geometry in patients with AN which is associated with a higher hip fracture risk [13, 14]. With aging there is a normal, physiologic decrease in bone microarchitecture, depicted by loss and widening of vertical and horizontal trabeculae, that leads to increased bone fragility. In a study of 57 girls with AN (mean age of 15.1), bone microarchitecture (calculated via trabecular bone score) was decreased in 40% of participants when compared to a healthy cohort [46]. Interestingly, the average BMI of these individuals was relatively high at 18.9 kg/m^2^ and was thought to be representative of a less severe AN population.

In multiple studies, bone turnover was found to be altered in patients with AN [30, 47, 48]. Bone turnover refers to the balance between bone resorption and bone formation as measured by markers from the peripheral blood or urine. Studies have found that bone formation markers are low and bone resorption markers are elevated in patients with AN including an increase in pyridinoline, deoxypyridinoline, N-telopeptide and C-telopeptide and a decrease in osteocalcin and total and bone-specific alkaline phosphatase [30, 47, 48]. Elevated turnover markers can still be detected during the initial 6–12 months of recovery from malnutrition but they do eventually normalize with continued nutrition and weight restoration [30, 47].

Finally, bone mineralization refers to the establishment of a mineral matrix within the bone after formation is complete. It includes the development of a collagen matrix as well as calcium and phosphate deposition within the bone. A study of 24 AN patients between 13 and 18 years of age showed significantly lower trabecular bone mineral content and volumetric bone mineral density of the forearm compared to age and height-matched adolescents [20].

The aforementioned studies demonstrate how low bone mineral density in AN results from multiple pathophysiologic processes. These data provide scientific plausibility for the drastically elevated fracture prevalence in patients with AN which is observed in both the short-term as well as in longstanding AN. In a study by Faje et al., 310 patients with active AN aged 12–22 were found to have an increased lifetime fracture prevalence that was 59.8% higher than age-matched comparisons. This fracture incidence peaked after the diagnosis of the disorder and occurred even at minimal reductions of BMD [49]. Long-term evaluations of bone health in AN suggest the severe and persistent nature of bone disease in this population [9, 10, 12, 43]. Decades after complete weight restoration, changes in bone density can still be seen, such as in a study from Switzerland demonstrating significantly reduced bone mass 27 years after complete disease recovery [43]. In another study, patients who had recovered from AN for as long as 21 years were still noted to have a decreased BMD [12]. These studies suggest that BMD in patients with AN never completely “catches up” or reaches the peak bone mass that otherwise would have been obtained [12, 43]. In fact, patients whose eating disorder developed in adolescence, which is the time of greatest bone mass accrual, are thought to be more prone to low BMD as near 90% of total bone mass is gained during this time [50].

An increased fracture risk has also been noted in patients with AN. One large study in Denmark evaluated 2149 AN patients and found a 2-fold increase in fracture risk 10 years after the initial diagnosis [10]. Another study found an increased fracture risk of approximately 3 standardized incidence ratio (SIR) for males and females 40 years after disease diagnosis [9]. Due to the retrospective nature of these studies, it is not known if these patients received treatment for low BMD during the course of their disease.

### Approach to diagnosis and management

The majority of studies regarding bone mineral density in the eating disorder population are limited by small study populations and narrow demographics, making clear recommendations challenging. For example, male subjects are rarely included in current studies but are an increasingly important presence in the eating disorder population [1]. Clear guidelines are urgently needed given the susceptibility of the typical, young eating disorder patient to lifelong debilitating fractures. Study reviews, expert opinion and rigorous peer-based evaluation would be an optimal approach for creating standards in this heretofore neglected field of eating disorder comorbidities.

### Diagnosis of low bone mineral density in eating disorders

A thorough medical and social history, physical exam and basic laboratory evaluation should be performed on all patients with AN suspected of having bone disease. Causes of secondary osteoporosis should be evaluated (Table 1) with special attention to medication use (especially steroids, diuretics, SSRIs, AEDs and depot medroxyprogesterone acetate). Basic lab work including a complete blood count, comprehensive metabolic panel, thyroid function tests, 25-hydroxyvitamin D and total testosterone in men are included in a baseline workup (Table 2). Initial DXA screening for children and adolescents is suggested if amenorrhea has been persistent for at least 6 months and serial screening is recommended yearly [51, 52]. In adults with AN, guidelines do not exist, however our current practice is to check a DXA on any male or female inpatient with an active eating disorder for 6 months or more. The frequency of follow-up screening is recommended every two years for adults according to the National Osteoporosis Foundation (NOF) [53], and in patients with worsening or persistent AN, repeat DXA may be indicated sooner if the results would change management. Patients with malnutrition as a result of ARFID or BN should also receive a screening DXA.

**Table 1:** Common causes of secondary osteoporosis

Premenopausal amenorrhea	
Hypercortisolism (i.e. Cushing Syndrome)	
Celiac disease, inflammatory bowel disease, short gut or other malabsorption syndrome	
Osteomalacia	
Liver or renal disease	
Low testosterone	
Low body weight or malnutrition	
Hyperthyroidism	
Rheumatoid arthritis, Systemic lupus erythematosus, connective tissue diseases or chronic inflammatory conditions	
Medications (i.e. steroids, diuretics, AEDs, depot medroxygprogesterone, SSRIs)	
Diabetes mellitus	
Vitamin D or calcium deficiency	
Current cigarette smoking	
Alcohol consumption, tobacco use

**Table 2:** Basic laboratory evaluation of osteoporosis

Serum chemistry panel (includes calcium, phosphorous, albumin, magnesium, liver function tests, alkaline phosphatase, creatinine)	
Complete blood count	
Thyroid stimulating hormone	
25-hydroxyvitamin D	
Testosterone panel in men	

For patients with eating disorders, general management recommendations are discussed below. For eating disorder patients that are peri- or postmenopausal or for males over the age of 50, diagnosis and management follow WHO diagnostic criteria and guidelines, which includes thorough history taking to exclude secondary etiologies, fracture risk assessment using FRAX and pharmacologic therapy if warranted [15, 17].

### Treatment of low bone mineral density in eating disorders

Aggressive management of the underlying eating disorder is the mainstay of therapy. The primary goals of this are weight restoration and resumption of spontaneous menses in females. Adequate intake of vitamin D and calcium should be ensured.

Meta-analyses and systematic reviews analyzing the efficacy of weight restoration in improving BMD show supportive results [28, 54–56]. BMD of the spine can increase up to 3.1% with weight gain although improvements may be slow and may not be detectable for up to 16 months [54]. In females, one study demonstrated that significant BMD improvement was only seen if the weight gain was substantial enough to result in resumption of spontaneous menses [57]. In addition to these supportive data, weight gain is the safest method of improving bone density in patients with AN, helps reverse any other concomitant disease complications and provides the foundation for sustained disease recovery.

Weight-bearing exercise and physical activity are generally recommended in non-eating disorder patients with low bone mineral density [58, 59]. Mechanical loading has osteogenic properties, can positively alter bone geometry and lead to increased bone mineral accrual in youth [60]. Physical activity can also reduce the risk of falls, which in turn decreases fragility fractures [61]. However, in patients with AN, recommendations are limited and study results vary based on the severity of illness and type of mechanical loading [62]. In a study from 2011, even moderate exercise was associated with lower lumbar and total body BMD in ill patients [62]. Exercise in patients with active AN may hasten weight loss and cause further complications associated with low body weight [62]. Adequate nutrition and the presence of spontaneous menses in females is thought to be protective to the bone as historically described by the concept of the Female Athlete Triad. This triad was modified in 2000 and is now described as a spectrum disorder with low energy availability (with or without disordered eating), menstrual dysfunction and low bone mineral density [63, 64]. Data comparing BMD in athletes and non-athletes are lacking but studies on amenorrheic and eumenorrheic athletes have shown that amenorrheic athletes have lower bone mineral density, lower estimated bone strength and abnormal bone microarchitecture compared to eumenorrheic athletes [65, 66]. Providers should make cautious, graded recommendations regarding exercise and weight-bearing activity based on the patient’s degree of recovery from their malnutrition.

Vitamin D and calcium stores should be optimized in patients with low BMD and vitamin D deficiency should be treated. Vitamin D is important as it enhances intestinal resorption of calcium and phosphorous, which is essential for establishment of the bone matrix. Conflicting data exist regarding optimal vitamin D and calcium stores, however, no specific studies have been conducted in the eating disorder population. There is no evidence of improved BMD with vitamin D and calcium alone in patients with AN, however, one study did show a strong negative linear relationship between 25OH-D levels in eating disorder patients and hip BMD [67]. Patients with eating disorders are known to have significantly lower serum 25OH-D and 1,25OH-D levels compared to healthy controls despite reportedly similar vitamin D intake [68]. The Endocrine Society defines vitamin D deficiency as a serum level < 20 ng/mL and insufficiency between 21 and 29 ng/mL. Patients with vitamin D deficiency should be treated with 50,000 international units (IU) of vitamin D (frequently ergocalciferol) weekly until serum levels are >30 ng/mL, followed by maintenance dosing for fracture prevention. Patients with vitamin D insufficiency can be started on 600-800 IU daily, however, may require doses between 1500 and 2000 IU daily if their level does not improve to >30 ng/mL. If patients have evidence of low bone mineral density but have normal vitamin D levels, they should still be started on daily maintenance vitamin D supplementation of 600-800 IU [69–71]. Approximately 1200 mg of calcium are suggested as the daily dosage for optimal bone health but recommendations change based on age and sex [71] (Table 3). Alimentary calcium tends to be better tolerated and absorbed than supplementation. If supplements are required, calcium carbonate is typically used unless the patient is on a proton-pump inhibitor (PPI) or H2-blocker, in which case calcium citrate is preferred [71].

**Table 3:** Daily Calcium Intake Reference (adapted from the Institute of Medicine 2010)

Age	Estimated Average Requirement of Calcium (mg/day)	Recommended Dietary Allowance of Calcium (mg/day)	Upper Level Intake of Calcium (mg/day)
14–18 years old	1100	1300	3000
19–30 years old	800	1000	2500
31–50 years old	800	1000	2500
51–70 year old males	800	1000	2000
51–70 year old females	1000	1200	2000

For adult, premenopausal patients with eating disorders who have persistent risk for BMD loss (i.e. active disease), a Z-score ≤ −2, a history or fractures or are at a high risk for fractures can be considered for pharmacologic therapy of osteoporosis. The abovementioned recommendations regarding weight restoration, resumption of spontaneous menses and repletion of vitamin D and calcium stores are prerequisites for further pharmacologic management and should be discussed at length with patients.

Specifically for patients with bone age ≥ 15 years, physiologic transdermal estrogen with oral progesterone can be considered for treatment of osteoporosis as it improved BMD in one study of female adolescents with AN [72]. Oral contraceptive pills (OCPs) are insufficient in improving BMD in AN due to a suspected estrogen-induced suppression of insulin-like growth factor I (IGF-I), which is a bone anabolic agent [28, 73, 74]. One study, however, did find a bone density increase of 4% with OCPs in a severely malnourished subset of patients who weighed <70% of their ideal body weight [73], yet these data have not been reproduced elsewhere. OCPs had positive effects on bone density in other populations of healthy, premenopausal women, but the evidence is conflicting and OCPs are therefore not recommended for this purpose [75, 76].

Testosterone is a known anabolic bone agent and is deficient in females and adolescent boys with AN [31, 77, 78]. One study in females with AN found that lean mass, but not BMD, increased with use of transdermal testosterone replacement [79]. Hypogonadism is a principal cause of osteoporosis in men without disordered eating and testosterone administration has been effective in increasing BMD in males with central and primary hypogonadism [80, 81]. For example, one study found an impressive spinal BMD increase of 5% and a 7% lean mass increase after 18 months of intramuscular testosterone therapy (100 mg/week) [80]. Most recently, transdermal testosterone treatment in older men with low testosterone significantly increased BMD after one year [82]. No studies have evaluated the prevalence of acquired hypogonadism in adult males with AN nor the possible efficacy of testosterone replacement in this population. Similar to estrogen, testosterone should not be used in patients with a bone age ≤ 15 years or in the presence of unfused epiphyses. If testosterone stores are replete and a high fracture risk exists due to ongoing AN, use of other pharmacologic agents could be considered for males.

Bisphosphonates improve bone mineral density by inhibiting osteoclast activity and reducing bone resorption and turnover. They become integrated into the bone matrix and due to their half-life of >10 years, their function is still noted long after treatment cessation. Bisphosphonates have been in use for over 20 years and have been studied in patients with AN. Different formulations of bisphosphonates appear to be equally effective [83] and, in postmenopausal women, they have been shown to reduce the risk of fracture by approximately 50% [84]. Two bisphosphonates (alendronate and risedronate) have been approved by the US Food and Drug Administration (FDA) for treatment of premenopausal osteoporosis in patients with steroid-induced osteoporosis and have also been shown to improve BMD in cases of osteoporosis caused by pregnancy, lactation, cystic fibrosis and thalassemia [85–88]. In eating disorder patients, two randomized controlled trials were performed showing efficacy of bisphosphonates (alendronate and risedronate) with an approximate BMD gain of 2–4.4% [79, 89]. The alendronate study, however, found weight restoration to be the most important determinant of BMD improvement at follow-up and BMD was higher at the femoral neck with alendronate only after correcting for change in body weight [89]. Bisphosphonates are classified as pregnancy risk factor C, can cross the blood-placenta barrier and lead to fetal hypocalcemia [90, 91]. In the typical young, female AN patient with the potential to regain fertility, contraception is recommended during and after treatment but their use is generally discouraged. Bisphosphonates have an appealing role in the treatment of male AN patients with osteoporosis given the irrelevance of teratogenic risk, however, this has not yet been studied.

Teriparatide is a recombinant 1–34 parathyroid hormone and an anabolic bone growth agent that stimulates preosteoblasts and calcium reabsorption in the kidneys. It is approved by the FDA for treatment of osteoporosis in adults and has been used in premenopausal women with steroid-induced osteoporosis with a high fracture risk [76] and in patients with idiopathic osteoporosis [92]. Given the black box warning of osteosarcoma, fusion of the epiphyses should be well documented in patients under the age of 25 [92]. One recent randomized controlled trial in eating disorder patients demonstrated a dramatic BMD increase of 6–10% [93]. Given the coupled nature of the bone remodeling properties of teriparatide, prolonged use (>24 months) leads to osteoclast stimulation and therefore bone resorption and loss of BMD. Teriparatide cessation without sequential antiresorptive therapy leads to loss of bone mass in postmenopausal women and declines by 4.8% at the lumbar spine in patients with premenopausal idiopathic osteoporosis [94]. In a randomized controlled trial of post-menopausal women using teriparatide (DATA-Switch study), sequential therapy with denosumab resulted in a continued increase of BMD after cessation of teriparatide [95]. It is likely that patients with AN would require antiresorptive therapy after teriparatide although this has not been evaluated. Long-term effects of teriparatide on the premenopausal population have not been studied and this medication should therefore be used with caution in these patients. Similar to bisphosphonates, teriparatide has a pregnancy risk factor C, however, no long term pregnancy-related risks have been noted.

Denosumab has not yet been studied in patients with eating disorders. However, denosumab is FDA approved for the treatment of post-menopausal osteoporosis [96]. It is a human monoclonal antibody that inhibits osteoclast activation via binding on the RANK ligand. There are limited studies on the use of denosumab in premenopausal women but it is an appealing option in younger populations given its ease of administration (one injection every six months) and shorter half-life compared to bisphosphonates (25.4 days). Denosumab is contraindicated in pregnancy and premenopausal women on this medication should be on contraception during and up to five months after completion of treatment. Patient consent of the risks and benefits of this medication should be well documented. Other long-term side effects on the premenopausal population have not been studied.

Romosozumab is a monoclonal antibody that binds and inhibits sclerostin and is a new, emerging option for osteoporosis management in post-menopausal women and may eventually have a role in the treatment of osteoporosis in eating disorder patients. Sclerostin is produced by osteocytes which inhibits bone formation and was found to be elevated in a cohort of females with AN [97, 98].

## Conclusion

There are currently no existing guidelines for the treatment of eating disorder patients with low bone mineral density. This review aims to provide a summary of the literature to date and current options for prevention and management, however, a standardized approach for low BMD in this patient population through rigorous peer-review is needed. Future studies are warranted regarding use of osteoporosis agents, such as denosumab, in patients with AN as well as evaluation of the long-term side effects of these medications in younger populations.

Based on the current literature, if a patient with AN has evidence of low BMD, weight restoration with resumption of spontaneous menses is the mainstay of therapy. Patients should be screened for causes of secondary bone mineral loss and a thorough history and physical exam should be performed. Baseline laboratory data should be obtained and vitamin D and calcium stores optimized. Weight-bearing exercise should be avoided initially but can be gradually reintroduced at the discretion of the treatment team if weight gain is achieved. Post-menopausal women or men over the age of 50 with an eating disorder should be managed per WHO recommendations [15]. Premenopausal women or men under the age of 50 who have a Z-score ≤ −2, history of or risk for fracture or ongoing bone mineral loss can be considered for use of pharmacologic osteoporosis agents as a bridge until full weight restoration occurs. Bone age should be greater than 15 years prior to initiating pharmacologic or hormonal treatment in younger patients.

Ultimately, deciding which pharmacologic agent to use is often a question of administration logistics and cost and should be discussed with the patient and tailored to their specific needs. Other factors, such as previous pharmacologic treatment, possibility of future pregnancy, disease severity, medication side effect profile, likelihood of medication compliance and follow up care, can affect selection of the appropriate agent. Evidence of efficacy in eating disorder patients has been demonstrated with the following: physiologic transdermal estrogen plus oral progesterone, bisphosphonates (alendronate or risedronate) and teriperatide. There are no definitive guidelines when treating osteoporosis in males with eating disorders, however, if concomitant hypogonadism is detected, treating with testosterone is reasonable. Denosumab has not been studied in premenopausal eating disorder patients but is an appealing option based on its ease of administration, shorter half-life and lack of skeletal accumulation. Further research is needed in this group of patients who are highly susceptible to rapid loss of bone mineral density to prevent fractures and potentially debilitating and irreversible deformities.

## Abbreviations

AEDs: Antiepileptic drugs
AN-BP: Anorexia nervosa-binge purge subtype
AN-R: Anorexia nervosa-restricting subtype
ARFID: Avoidant/restrictive food intake disorder
BAT: Brown adipose tissue
BMD: Bone mineral density
BMI: Body mass index
BN: Bulimia nervosa
D2: Ergocalciferol
D3: Cholecalciferol
DSM – V: Diagnostic and Statistical Manual of Mental Disorders, Fifth Edition
DSM-IV: Diagnostic and Statistical Manual of Mental Disorders, Fourth Edition
DXA: Dual-energy X-ray absorptiometry
ED-NOS: Eating disorder not otherwise specified
FDA: US Food and Drug Administration
FRAX: Fracture risk assessment tool
GH: Growth hormone
IGF-1: Insulin-like growth factor-1
IOF: International Osteoporosis Foundation
ISCD: International Society of Clinical Densitometry
NOF: National Osteoporosis Foundation
OCP: Oral contraceptive pills
PPIs: Proton-pump inhibitors
PTH: Parathyroid hormone
RANKL: Receptor activator of nuclear factor kappa-B ligand
SIR: Standardized incidence ratio
SSRIs: Selective serotonin reuptake inhibitors
WHO: World Health Organization
